# Severity of COVID-19 in Patients with Diarrhoea: A Systematic Review and Meta-Analysis

**DOI:** 10.3390/tropicalmed8020084

**Published:** 2023-01-26

**Authors:** Sunita Dhakal, Pimphen Charoen, Wirichada Pan-ngum, Viravarn Luvira, Chaisith Sivakorn, Borimas Hanboonkunupakarn, Sakkarin Chirapongsathorn, Kittiyod Poovorawan

**Affiliations:** 1 Department of Clinical Tropical Medicine, Faculty of Tropical Medicine, Mahidol University, Bangkok 10400, Thailand; 2 Department of Tropical Hygiene, Faculty of Tropical Medicine, Mahidol University, Bangkok 10400, Thailand; 3 Department of Gastroenterology and Hepatology, Phramongkutklao Hospital, College of Medicine, Bangkok 10400, Thailand

**Keywords:** nCoV COVID-19, gastrointestinal tract, diarrhoea, SARS-CoV-2, nausea, vomiting, abdominal pain, anorexia

## Abstract

COVID-19 patients occasionally present with diarrhoea. Our objective was to estimate the risk of developing the severe disease in COVID-19 patients with and without diarrhoea and to provide a more precise estimate of the prevalence of COVID-19-associated digestive symptoms. A total of 88 studies (n = 67,794) on patients with a COVID-19 infection published between 1 January 2020 and 20 October 2022 were included in this meta-analysis. The overall prevalence of digestive symptoms was 27% (95% confidence interval (CI): 21–34%; I^2^ = 99%). According to our data, the pooled prevalence of diarrhoea symptoms in the 88 studies analysed was 17% (95% CI: 14–20%; I^2^ = 98%). The pooled estimate of nausea or vomiting in a total of 60 studies was 12% (95% CI: 8–15%; I^2^ = 98%). We also analysed 23 studies with eligible individuals (n = 3800) to assess the association between the disease severity and diarrhoea. Individuals who had diarrhoea were more likely to have experienced severe COVID-19 (odds ratio: 1.71; 95% CI: 1.31–2.24; *p* < 0.0001; I^2^ = 10%). Gastrointestinal symptoms and diarrhoea are frequently presenting COVID-19 manifestations that physicians should be aware of.

## 1. Introduction

Coronavirus disease 2019 (COVID-19) is the fifth pandemic after 1918 H1N1 (Spanish flu), 1957 H2N2 (Asian flu), 1968 H3N2 (Hong Kong flu) and 2009 H1N1 (Influenza) [1]. The disease was discovered in China, and its most common clinical symptoms are fever, cough, sore throat, rhinorrhoea, headache, fatigue, shortness of breath, abdominal pain and anosmia [2]. However, a small percentage of patients experience gastrointestinal symptoms, with diarrhoea being the most common symptom and thought to be present in 10–50% of the infected population [3,4]. The gastrointestinal tract is believed to be affected because of a direct viral invasion mediated by the binding of the virus to the angiotensin-converting enzyme-2 (ACE-2) receptor, thereby causing cytotoxic damage [5].

Emerging data indicate that diarrhoea is associated with severe COVID-19 and suggest that diarrhoea could be a reliable indicator of the onset of severe COVID-19 [6]. In other studies, diarrhoea has been linked to an increase in the severity of the COVID-19-associated pathology [7]. Although clinical research comparing COVID-19 in the presence and absence of diarrhoea symptoms has already been conducted, significant conclusive evidence has yet to surface, and most studies have been conducted on a small sample size.

Herein, we attempted to better understand the relationship between COVID-19 and gastrointestinal symptoms by comparing the risk of developing the severe disease in COVID-19 patients with diarrhoea, the most common gastrointestinal manifestation. We analysed various data points related to disease severity with diarrhoea and other gastrointestinal manifestations in COVID-19 patients by using a systematic review and a meta-analysis of previously published studies in order to generalise the correlation across different settings. According to numerous studies, patients with severe COVID-19 are more likely to develop diarrhoea than are patients with non-severe COVID-19. However, because of inconsistencies in the analysed results and a lack of data, the exact effect of diarrhoea on COVID-19 remains unknown. As a result, additional research on the prevalence of gastrointestinal manifestations and the diarrhoea-associated severity in COVID-19 patients is required.

## 2. Materials and Methods

On 7 June 2021, our protocol was registered with PROSPERO, the International Prospective Register of Systematic Reviews (registration number: CRD42021234776). The criteria for the studies’ inclusion and exclusion are listed in Table 1.

### 2.1. Methodology of Search and Selection Criteria

A systematic review and a meta-analysis were performed to assess the severity of the COVID-19 infection in patients with diarrhoea versus those without diarrhoea, by comparing severely and non-severely ill patient groups. We searched reputed databases for articles published between 1 January 2020 and 20 October 2022 by using the medical subject heading and the keywords ‘nCoV’, ‘SARS-CoV-2′, ‘digestive system’, ‘diarrhoea’, ‘abdominal pain’, ‘nausea’, ‘vomiting’, ‘anorexia’, ‘favipiravir’, ‘lopinavir/ritonavir’, ‘antibodies’, ‘monoclonal’, ‘molnupiravir’, ‘Sequential Organ Failure Assessment (SOFA)’, ‘APACHE II’, ‘hyponatraemia’, ‘hypokalaemia’, ‘intensive care unit (ICU)’, ‘SARS-CoV-2 variants’ and ‘COVID-19 vaccines’.

We searched PubMed, Scopus, Embase, Cochrane Library, ProQuest and the WHO publications’ database. A secondary search was undertaken by using published study references. References from all studies were checked for any additional sources of knowledge. Only peer-reviewed, English-language studies were included in our search, and only those studies that had been accepted for publication were considered. The online Rayyan Systematic Review platform was used to manage all relevant articles, including duplicates. We included studies that reported the severity of diarrhoea in infected patients and excluded studies with no availability of diarrhoea data, no full-text articles, duplicate publications, review articles, studies with an identical population, case series and case reports.

### 2.2. Extraction of Data and Definitions

Two independent reviewers (SD and KP) screened the titles and abstracts that met the eligibility criteria. We obtained the full-text articles of the studies that passed our initial screening of titles and abstracts. Subsequently, the full-text of the remaining articles were reviewed to see if these articles satisfied the inclusion criteria and were appropriate for further analysis. When dissonance between reviewers occurred, the complete text and extracted data were further reviewed by a third reviewer (BH) to corroborate validity. Data extraction was performed by employing a pre-defined form that included the following data: author, date and year of publication, study design (cohort, cross-sectional, case–control, prospective and case series studies), country, sample size, patient demographics, vital signs, both participants with severe and those with non-severe diarrhoea, the prevalence of digestive manifestations (e.g., diarrhoea, abdominal pain, nausea, vomiting and loss of appetite), associated comorbidities (e.g., hypertension, cardiovascular disease, diabetes and neurological disease), Acute Physiology and Chronic Health Evaluation II (APACHE II) score, duration of ICU stay, SOFA score, hypokalaemia, hyponatraemia, COVID-19 variant associations, vaccine administration and use of antibiotics and antiviral treatments.

Severe COVID-19 infection was defined as the onset of more severe signs and symptoms 1 week after the onset of the first symptoms, an ICU admission for mechanical ventilation as required for patients with a respiratory rate of ≥30 breaths per minute and an oxygen saturation (SpO_2_) ≤93% and for patients with lung infiltrates of ≥50% resulting in dyspnoea, a ratio of arterial oxygen partial pressure to fractional inspired oxygen (PaO_2_/FiO_2_) of <300 mm Hg [8], higher APACHE II and SOFA scores, an electrolyte imbalance (manifesting as hyponatraemia and hypokalaemia) and the use of antiviral drugs (e.g., lopinavir/ritonavir) causing diarrhoeal side effects. This study followed the recommendations outlined by the PRISMA guidelines.

### 2.3. Assessment of the Risk of Bias

Each study’s quality was determined by using inclusion and exclusion criteria and through grading (as good, fair or poor) by assigning stars to each domain according to the Newcastle–Ottawa Scale (NOS) guidelines [9]. Eight items were examined in total, divided into three subscales that are classified as ‘selection’, ‘comparability’ and ‘outcome/exposure’ in NOS case–control and cohort studies, and the total maximum score that can be assigned for those three subsets was 9 [9]. A study receiving a score of ≥7 was judged to be of ‘high quality’ or ‘good’. The mean value of the 17 cohort studies was 6.5 (Table S1). As a result, we had two case–control studies with a mean value of 8 that were assessed as high-quality studies, and one cross-sectional study that was considered satisfactory (Table S2 and Table S3). Similarly, the National Institutes of Health (NIH) Quality Assessment Tool for Case Series Studies) [10] was used for the assessment of case series studies; this tool examines nine elements. In the case of the three included studies, an assessment was conducted, and the studies were found to be of good quality (Table S4). Disagreements between assessments were settled following a discussion amongst the reviewing authors (SD and KP). When any additional disagreements arose, these were resolved through consultation with the third reviewing author (BH).

### 2.4. Outcome

Our primary outcome analysis compared the severity of the COVID-19 infection in patients with diarrhoea versus those without diarrhoea. Secondary outcomes were used to estimate the prevalence of the gastrointestinal symptoms in COVID-19 patients.

### 2.5. Statistical Analysis

A meta-analysis was conducted to combine the effect sizes of all included studies. software (R software version 4.1.1; R Foundation, Viena, Austria; meta, dmetar and metafor) was used to estimate the prevalence of gastrointestinal symptoms, while RevMan 5.3 (RevMan software version 5.3. Copenhagen: The Nordic Cochrane Centre, The Cochrane Collaboration) was used for the assessment of the severity of COVID-19 in patients with diarrhoea; severely versus non-severely ill patient groups were compared. In an attempt to estimate the prevalence of the COVID-19-associated gastrointestinal symptoms, we extracted demographic data from relevant studies, sample sizes and events. Firstly, we used inverse variance to estimate the weight of the individual studies. Subsequently, the Freeman Tukey double arcsine transformation [11] was applied, thereby giving more weight to studies with a higher value than to those with a lower value. A random-effects model and an odds ratio (OR) with a 95% confidence interval (CI) were used for the assessment of the severity of COVID-19 in a patient with diarrhoea between severely and non-severely ill patient groups. To calculate the statistical heterogeneity, I^2^ and Cochrane’s Q test were utilised. The Forest plots represented the combined effects, while the OR was considered statistically significant when the *p*-value was < 0.05. Finally, funnel plots’ creation and the performance of Egger’s tests were facilitated through R software (R software version 4.1.1; R Foundation, Viena, Austria; meta, dmetar and metafor).

## 3. Results

**Figure 1:**
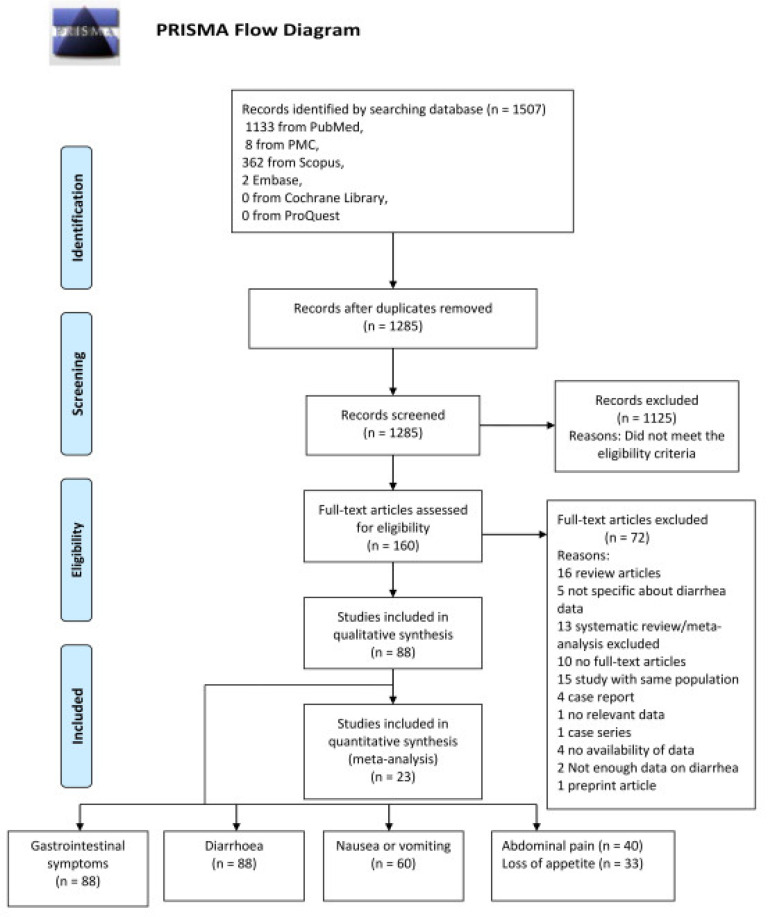
PRISMA flow diagram.

A total of 1507 records were identified. After removing duplicates, 1285 records remained. After screening for titles and abstracts, 160 articles were chosen for a full-text review; of these, 72 were retrieved for further assessment. Out of the 72 articles reviewed, 16 were excluded as review articles, 15 were excluded for examining the same cohort, 13 were excluded for being systematic reviews and meta-analyses, 10 were excluded for not being full-text articles, 5 were excluded for not providing specific diarrhoea-related data, 4 were excluded for being case reports or not providing data, 2 were excluded for not providing enough data and 1 was excluded for being a case series study with no relevant data. The remaining 88 studies comprised the qualitative synthesis, while the meta-analysis included 23 studies totalling 3800 COVID-19 patients (Figure 1).

### Study Characteristics and Statistical Findings

Amongst the analysed 88 studies [6,12,13,14,15,16,17,18,19,20,21,22,23,24,25,26,27,28,29,30,31,32,33,34,35,36,37,38,39,40,41,42,43,44,45,46,47,48,49,50,51,52,53,54,55,56,57,58,59,60,61,62,63,64,65,66,67,68,69,70,71,72,73,74,75,76,77,78,79,80,81,82,83,84,85,86,87,88,89,90,91,92,93,94,95,96,97], a total of 67,794 patients with COVID-19 infection were reported (Table 2). The countries of origin of these studies were the following: mainland China (n = 56), USA (n = 9), South Korea (n = 5), Japan (n = 2), Mexico (n = 2), Brazil (n = 1), Ethiopia (n = 1), Singapore (n = 1), Thailand (n = 1), Iraq (n = 1), France (n = 1), Italy (n = 1), Hong Kong (n = 1), Macau (n = 1), Iran (n = 2), Turkey (n = 1), Morocco (n = 1) and Malaysia (n = 1). Except for two prospective studies, most of the aforementioned studies were retrospective, with some being case–control and case series studies [33]. In COVID-19 patients, diarrhoea, abdominal pain, nausea, vomiting and loss of appetite were the most frequently reported gastrointestinal symptoms.

After pooling data from these 88 studies, the prevalence of gastrointestinal symptoms in COVID-19 patients was estimated to be 27.89% (95% CI: 21.97–34.22%; I^2^ = 99%) (Figure S1). Following that, the prevalence of diarrhoea was estimated to be 16.93% in these 88 studies (95% CI: 14.13–19.91%; I^2^ = 98%) (Figure S2). The first case of COVID-19 that was associated with diarrhoea, the most commonly associated gastrointestinal symptom, was reported in China [98]. Similarly, the pooled estimate of nausea and vomiting in 60 studies was 11.93% (95% CI: 8.86–15.37%, I^2^ = 98%) (Figure S3). According to our analysis, the prevalence of abdominal pain was 8.89% (CI 5.25–13.30%, I^2^ = 98%) in 40 studies (Figure S4), and the pooled estimate of a loss of appetite was 25.13% (CI 16.18–35.26%, I^2^ = 99%) in 33 studies (Figure S5).

In 23 out of the examined 88 studies, [6,15,19,23,27,30,33,34,40,43,51,54,66,69,79,81,82,83,88,92,93,94,95,96,97] a COVID-19 severity assessment in patients with diarrhoea was reported. Most of the studies were conducted in China, though four papers from other countries (namely, South Korea, USA, Thailand and Singapore) have also provided such assessments. Our analysis included 3800 patients who had COVID-19. The majority of these studies compared the severely versus the non-severely ill patients, [6,15,19,27,30,34,40,43,81,82,83,88,92,93,94,96] while others compared the ICU-admitted versus the non-ICU-admitted patients [23,33,54,66]. Moreover, one study reported on patients with an SpO_2_ of <90% versus patients with an SpO_2_ of >90% [69], and another reported on patients with their symptoms’ onset being ≤10 days versus patients with their symptoms’ onset being of ≥10 days (Table 3) [79].

**Figure 2:**
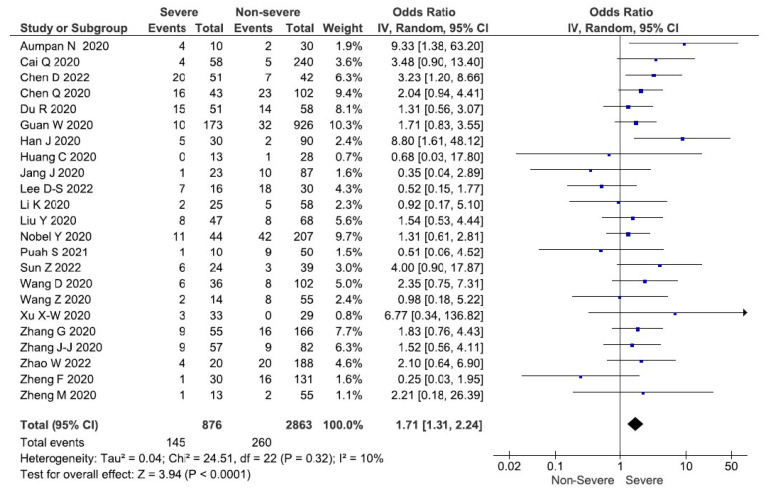
Forest plot demonstrating that in COVID-19 patients who have diarrhoea is associated with more severe COVID-19 outcome when the pooled odds of patients with non-severe diarrhoea versus severe diarrhoea are compared.

To synthesise the findings of these 23 studies, a meta-analysis using an inverse variance and a random-effects model was undertaken. The meta-analysis revealed that patients with COVID-19 and diarrhoea had an OR of 1.71 (95% CI: 1.31–2.24%; *p* < 0.0001) and as a result, a higher correlation with becoming severely ill. However, a minimal heterogeneity was detected amongst these studies (*χ^2^* = 24.51, df = 22; *p* = 0.32, I^2^ = 10%) (Figure 2). A funnel plot of the included studies highlights the publication bias. The funnel plot appears to be symmetrical. Moreover, an Egger’s test was used to verify the symmetry of the funnel plot. The results showed that there is no asymmetry in the plots (t = −0.02, df = 21; *p* = 0.9863) for the COVID-19 patients with diarrhoea, a finding that suggests that there is no publication bias (Figure S6).

## 4. Discussion

Patients with COVID-19 who present with diarrhoea have a higher risk of presenting with severe COVID-19. Several studies have found that the COVID-19 infection can cause gastrointestinal symptoms. Depending on the size of the sample and the number of the study sites involved, the prevalence and severity of these COVID-19-associated gastrointestinal symptoms vary. In this study, the severity assessment of COVID-19 patients with diarrhoea was compared between the severely and the non-severely ill patient groups, and the overall prevalence of COVID-19-associated gastrointestinal symptoms was computed. Out of 88 studies, 23 studies with 3800 COVID-19 patients were included for the undertaking of the meta-analysis for the delivery of the severity assessments. The chance of having a severe COVID-19 infection with diarrhoea was 1.71 (95% CI: 1.31–2.24%; *p* < 0.0001, I^2^ = 10%) times higher than those for the non-severely ill patient groups. According to the subgroup analysis, there are no significant differences in terms of value between Asian and non-Asian countries (Figure S7). As a result, we may infer that in individuals infected with COVID-19 and diarrhoea were more likely to be severely ill than those COVID-19 patients without diarrhoea. According to studies, changes in the microbiome of the gut are associated with a bidirectional shift in the interaction between the gut and a number of major organs, leading to severe disease symptoms explaining the gut–lung axis link in COVID-19 [99].

Patients with advanced age, obesity, hypertension, diabetes and dyspnoea are more likely to have severe disease [19,34,40,82]. Our meta-analysis for the severity evaluation has focused on the above risk factors. A study by Li et al., has reported that 28% of the severely ill group of COVID-19 patients had diabetes mellitus, 8% had hypertension, and 28% experienced dyspnoea [40]. This shows the relationship between comorbidities and COVID-19 severity. Moreover, studies have shown that male patients have a higher potential for developing severe COVID-19 than female patients [100,101]. After analysing the 23 included studies for severely ill COVID-19 patients with diarrhoea, our findings reveal that the pooled prevalence for male and female patients is 52.18% (95% CI: 48.67–55.67%; *p* < 0.01; I^2^ = 70%) and 47.83% (95% CI: 44.34–51.34%; *p* < 0.01; I^2^ = 70%), respectively. Hence, further analysis (that would include more data) must be performed to determine the role of sex in the severity of COVID-19.

Other parameters associated with the development of severe COVID-19 included an SpO_2_ of <92%, higher APACHE II and SOFA scores, bilateral lung infiltrates, decreased lymphocytes, ICU admission and prolonged hospitalisation [19,23,34,81]. In a study of 43 highly infected patients, APACHE II and SOFA scores were found to be greater in critically ill patients [19]. Further research is required to clarify the relationship between these parameters in severe COVID-19 patients with diarrhoea.

We analysed the overall prevalence of gastrointestinal symptoms in COVID-19 patients and found it to be 27%. Based on multiple studies, the most commonly reported gastrointestinal symptoms were diarrhoea, nausea, vomiting, abdominal pain and loss of appetite [52]. According to our findings, the overall prevalence of diarrhoea was 16%. However, the prevalence of diarrhoea ranged from 10% to 45% in various studies [37,102]. Diarrhoea can be the primary complaint, or it can be accompanied by fever. In a study of 103 individuals, Pan et al. reported six patients with digestive symptoms but no respiratory involvement, while 97 patients had both a digestive and respiratory involvement [52].

According to Jin et al., diarrhoea is defined as the passing of loose stool more than three times a day or as having an average of three evacuations per day [36]. Diarrhoea begins 1 to 8 days after the onset of the illness, with a median of 3.3 days; its duration ranges from 1 to 14 days, with an average of 4.1 ± 2.5 days. The maximum number of diarrhoea episodes per day is nine, while the average frequency is 3.3 ± 1.6 times per day, with 34.3% of those being watery stools [103]. There is no mucus present in the stools [104]; however, significant severe gastrointestinal symptoms are associated with blood [105]. Gastrointestinal bleeding is one of the complications of COVID-19 infection, which can possibly be confused with diarrhoea in some clinical contexts. In a study by Xiao et al., the stool features were described as yellow with no erythrocytes or leukocytes [77]. In contrast, in a study by Fang et al., leukocytes in the stool were identified in 3 out of 58 patients [103]. Owing to the disparity in results, more research is required to gain a better understanding of the stool characteristics and of their association with severe COVID-19 accompanied by diarrhoea.

Studies have also reported that symptoms are longer in diarrhoea COVID-19 patients than in those who do not have diarrhoea. As a result, it takes a longer time to eliminate SARS-CoV-2 in these patients, thereby resulting in longer hospital stays and an increased susceptibility to faecal–oral transmission [71]. According to a study conducted by Wei et al., COVID-19 patients with diarrhoea have a higher SARS-CoV-2 RNA load in their stool than those without diarrhoea [71]. A high viral load in the stool and a prolonged hospitalisation also suggest that COVID-19 patients with diarrhoea are more prone to be severely ill [71]. Several hypotheses have been proposed in an attempt to explain the occurrence of diarrhoea in COVID-19 patients [106].

According to recent review articles by Wang et al., diarrhoea could have been caused by direct viral invasion, resulting in cytotoxic damage after binding to ACE2 receptors. The attaching of SARS-CoV-2 to the ACE2 receptor can cause an ACE2 downregulation, that in turn causes a sodium-dependent glucose transport dysregulation, thereby resulting in significant gastrointestinal tract injury. An increase in proinflammatory mediators and cytokine storms may damage the digestive tract [5]. In COVID-19 patients, SARS-CoV-2 infection can cause gut dysbiosis by altering the intestinal microbiota which affect gut microbiota composition [5].

The COVID-19-associated gastrointestinal symptoms include nausea and vomiting, in addition to diarrhoea. According to our findings, 11.93% of COVID-19 patients experience nausea or vomiting. Similarly, 8% of these people experience abdominal pain, and 27% report a loss of appetite.

Several antiviral medications, such as lopinavir/ritonavir, may have contributed to this COVID-19-associated diarrhoea. According to one study, antiviral treatments for diarrhoea were ameliorated and subsequently stopped altogether [107]. In another trial, lopinavir and ritonavir did not affect the development of diarrhoea [108]. In this analysis, we looked at the use of lopinavir/ritonavir in severely infected COVID-19 individuals reported in six studies. The pooled estimate of the use of lopinavir/ritonavir in 743 severely infected COVID-19 individuals was 67.24% (95% CI: 27.38–96.33%; *p* < 0.01; I^2^ = 99%). Antiviral usage is fairly prevalent in patients with severe COVID-19; hence, more research is needed to establish if antivirals are linked to the development of diarrhoea in severe COVID-19 patients.

Recently, favipiravir has been linked to diarrhoea. According to the WHO database, 7% of 93 people who took favipiravir had diarrhoea [109]. In one of the herein analysed studies, favipiravir was used for the treatment of severe COVID-19 infections [15]. Remdesivir, in contrast, was not included in any of the studies. However, remdesivir has been linked to diarrhoea in 9% of the patients receiving it [110]. Additional research is needed to determine whether these antivirals are associated with severe COVID-19-associated diarrhoea. Other drugs, such as molnupiravir (a novel antiviral medication), have been linked to the development of headaches and diarrhoea [111]. In both the placebo and molnupiravir-receiving groups, multiple molnupiravir doses were associated with a 7.1% chance of developing diarrhoea [112]. The use of monoclonal antibodies in the form of a bamlanivimab monotherapy for the treatment of COVID-19 has been shown to cause nausea and diarrhoea in 1% of those receiving 700 mg, in 1.9% of those receiving 2800 mg and in 5.9% of those receiving 7000 mg; in contrast, combination therapy of bamlanivimab with etesevimab has been reported to cause diarrhoea in 1% of the patients receiving it [113]. Additional research on these novel antiviral treatments and monoclonal antibodies will be needed in the future to gain a better understanding of their role in generating diarrhoea in severely ill COVID-19 patients.

There were 23 studies that were included in our analysis. There was no information on vaccine administration in any of these trials. According to our findings, one study was linked to the B1.1.7 variant epidemic [54]. Aside from fever, cough and sore throat, B1.1.7 variants are known to trigger gastrointestinal problems in a limited number of people. European studies have reported diarrhoea and abdominal pain with the B1.1.7 variant [114]. In one particular study, the B1.6.1.7 (delta strain) was shown to cause abdominal pain, nausea, vomiting and diarrhoea [115]. In a study by Wang et al., 17 out of 25 critically ill COVID-19 patients experienced diarrhoea when infected with the delta strain [107]. However, of the 38 individuals who presented with severe symptoms, only 8 were found to be infected with the delta strain. None of the patients in the Wang et.al study had diarrhoea, but one in eight (twelve) had nausea and vomiting [116]. Because of the small sample size, it is difficult to establish whether there is a link between diarrhoea and specific COVID-19 variants. Based on past research, we may conclude that COVID-19 mutations are linked to gastrointestinal problems. The undertaking of further study is required to determine the degree of disease severity with these variants (including the omicron variant) in COVID-19; however, no relevant reports on the omicron variant have been published so far.

### 4.1. The Implications of the Study

COVID-19 individuals who experienced diarrhoea had an increased likelihood of being severely ill. This might be due to the existence of a gut–lung axis. Similarly, COVID-19 patients presented with gastrointestinal symptoms (27%) and diarrhoea (16%). Approximately 35% of those who have COVID-19 had diarrhoea as their first symptom, with no respiratory involvement. These findings aid physicians in raising awareness of the gastrointestinal involvement during the COVID-19 outbreak. The severity of COVID-19 is also related to the individual’s age and other comorbidities. The use of both new and old antiviral drugs can produce diarrhoea in most of patients, but further study is required to determine the relationship between antiviral usage and the severity of COVID-19-associated diarrhoea. The development of diarrhoea has also been linked to the use of monoclonal antibodies, while a variety of genetic mutations has also been associated with the development of diarrhoea.

### 4.2. Limitation of the Study

Our study has certain limitations; our research includes only papers written in English. There are several excluded articles that lack data on the disease severity and diarrhoea. The majority of the data included in the meta-analysis were from Asian countries. There was also a lack of data on antiviral drugs that may have influenced the disease severity, as well as a lack of data regarding the association of the undertaken vaccination and certain variants with diarrhoea and disease severity.

## 5. Conclusions

Our meta-analysis provides further evidence to support the hypothesis that the risk of developing severe COVID-19 in patients with diarrhoea is higher than in COVID-19 patients who did not have diarrhoea based on the study data during the COVID-19 pandemic. COVID-19 patients with gastrointestinal symptoms account for 27% of the cases, with diarrhoea being a symptom in 16% of COVID-19 patients. Physicians should also raise awareness that diarrhoea in COVID-19 might be associated with a more severe clinical course, and some COVID-19 patients might be presented with gastrointestinal symptoms. During the COVID-19 pandemic, clinicians should consider COVID-19 as a possible diagnosis for cases with gastrointestinal symptoms. Finally, clinical observation and early medical treatment should be prioritised when a patient is diagnosed with COVID-19-associated diarrhoea.

## Figures and Tables

**Table 1:** Table outlining the criteria applied for the herein undertaken study inclusion and exclusion.

Inclusion Criteria	Exclusion Criteria
Studies that included case-controls, cohorts, cross-sectional and prospective studies	Children, adolescents and pregnant women
Literature search on electronic databases, namely PubMed, PubMed Central, Embase, Scopus, Cochrane library and ProQuest (from 1 January 2020 and last updated on 20 October 2022) by using search terms that included ‘SARCOV-2′, ‘COVID-19′, ‘gastrointestinal symptoms’, ‘diarrhoea’ and ‘nCoV’	Articles that did not provide information on gastrointestinal symptoms
Articles reporting gastrointestinal symptoms associated with the COVID-19 infection that included diarrhoea, nausea, vomiting, abdominal pain and loss of appetite	Case reports, preprints, no full-text articles and no availability of the diarrhoea-associated data
Studies comparing severe versus non-severe diarrhoea	No relevant data
A patient who met the definition of severe disease as follows: (i) dyspnoea present, (ii) a respiratory rate: 30 or >breaths per minute, (iii) blood oxygen saturation: 93% or less, (iv) ratio of the partial pressure of arterial oxygen to the fraction of inspired oxygen (PaO_2_:FiO_2_): <300 mm Hg, (v) infiltrates in more than 50% of lung field, (vi) patient under mechanical ventilation and ICU (intensive care unit) admitted, (vii) APACHE II and SOFA scores higher for critically ill patients with COVID-19 infection, (viii) hyponatraemia or hypokalaemia	

**Table 2:** Characteristics of 88 studies: summarised overview of their demographic data, epidemiology and clinical outcomes.

Authors	Date/Year of Publication	Study Design	Country	Sample Size N (%)	Age, Mean ±SD/Median (IQR)	Male N (%)	Female N (%)	GI Symptoms N (%)	Diarrhoea N (%)	Abdominal Pain N (%)	Nausea or Vomiting N (%)	Loss of Appetite N (%)
Chang D, et al. [17]	March 2020	Case series	China	11	34 (34–48)	10	NA	1 (7.6)	1 (7.7)	NA	NA	NA
Xiong Y, et al. [74]	22 February 2020	Retrospective	China	35	49.5 ± 14.1	25 (60)	NA	10/42 (24)	10 (24)	NA	NA	NA
Liu K, et al. [42]	29 January 2020	Retrospective	China	137	57 (20–83)	61 (44.5)	76 (55.5)	11 (8.0)	11 (8.0)	NA	NA	NA
Guan W, et al. [27]	28 February 2020	Multicentre	China	1099	47.0 (35.0–58.0)	640	459/1096 (41.9)	≥55 (5.0)	42 (3.8)	NA	55(5.0)	NA
Han C, et al. [29]	31 March 2020	Retrospective	China	206	62.5 (27–92)	91	115	117 (56.7)	67 (32.5)	9 (4.4)	24 (11.7)	102 (49.5)
Huang C, et al. [33]	24 January 2020	Prospective	China	41	49.0 (41.0–58.0)	30 (73)	11 (27)	1 (3.0)	1/38 (3)	NA	NA	NA
Jin X, et al. [36]	24 March 2020	Retrospective	China	74	46.14 ± 14.19	37 (50.0)	74	74 (100)	53 (71.62)	NA	NA	NA
Liu Y, et al. [44]	9 February 2020	Case series	China	12	54.34 ± 18.011	8 (66.6)	4 (33.33)	2 (16.67)	2 (16.67)	NA	2 (16.67)	NA
Luan Y, et al. [46]	9 July 2020	Retrospective	China	117	61.9 ± 17.9	62 (53.0)	55 (47.0)	≥8 (6.87)	8 (6.8)	1 (0.9)	5(4.2)	8 (6.8)
Luo S, et al. [48]	20 March 2020	Retrospective	China	183	53.8	102 (56)	81 (44)	183 (100)	68 (37)	45 (25)	37 (20)	180 (98)
Ng Y, et al. [50]	13 February 2020	Retrospective	China	21	56 (37–65)	13 (62)	8 (38)	2 (9.5)	2 (10)	NA	NA	NA
Pan L, et al. [52]	14 April 2020	Cross- sectional	China	204	52.91 ± 15.98	107 (52.45)	97 (47.54)	103 (50.04)	35 (33.98)	2 (1.94)	4 (3.88)	81 (78.64)
Shi S, et al. [60]	25 March 2020	Retrospective, cohort	China	416	64 (21–95)	205 (49.27)	211 (50.7)	16 (3.8)	16 (3.8)	NA	NA	NA
Shi H, et al. [59]	24 February 2020	Retrospective	China	81	49.5 (11.0)	42 (52)	39 (38)	4 (4.9)	3 (4)	NA	4 (5)	1 (1)
Song F, et al. [63]	6 February 2020	Retrospective	China	51	49 ± 16	25 (49)	26 (51)	5 (9.82)	5 (10)	NA	3 (6)	9 (18)
An P, et al. [13]	6 February 2020	Retrospective	China	9	35.8 (28–45)	4 (44.44)	5 (55.56)	9 (100)	1 (11.1)	0	2 (22)	6 (66.7)
Li K, et al. [40]	29 February 2020	Retrospective	China	83	45.5 ± 12.3	44 (53.0)	39 (47.0)	7 (8.4)	7 (8.4)	7 (8.4)	NA	NA
Wang D, et al. [66]	7 February 2020	Retrospective, case series	China	138	56 (42–68)	75 (54.3)	63 (45.7)	55 (39.9)	14 (10.1)	3 (2.2)	5 (3.6)	55 (39.9)
Wang Z, et al. [69]	16 March 2020	Retrospective	China	69	42.0 (35.0–62.0)	32 (46)	37 (54)	10 (14.49)	10 (14)	NA	3 (4)	7 (10)
Wu J, et al. [73]	29 February 2020	Retrospective	China	80	46.1 ± 15.42	39 (48.75)	41 (51.25)	1 (1.25)	1 (1.25)	NA	1 (1.25)	NA
Xia P, et al. [75]	31 September 2020	Retrospective, cohort	China	81	66.6 ± 11.4	54 (66.7)	27 (33.3)	26 (32.1)	20 (24.7)	NA	8 (9.9)	26 (32.1)
Xiao F, et al. [76]	3 March 2020	Case series	China	73	43 (0.83–7)	41 (56.16)	32 (43.83)	26 (35.61)	26 (35.61)	NA	NA	NA
Xu X-W, et al. [79]	19 February 2020	Retrospective, case series	China	62	41 (32–52)	35 (56)	27 (44)	3 (8)	3 (8)	NA	NA	NA
Xu X, et al. [78]	28 February 2020	Retrospective	China	90	50 (18–86)	39 (43)	51 (57)	5 (6)	5 (6)	NA	5 (6)	NA
Zhang J, et al. [83]	19 February 2020	Retrospective	China	140	57 (25–87)	71 (50.7)	69 (49.3)	55/139 (39.6)	18/139 (12.9)	8/139 (5.8)	24/139 (17.3)	17/139 (12.2)
Zhang P, et al. [84]	4 June 2020	Retrospective	China	136	69 (57–77)	86 (63)	50 (37)	28 (21.0)	28 (21.0)	NA	NA	NA
Zhao W, et al. [86]	3 March 2020	Retrospective	China	101	44.44 (17–75)	56 (55.4)	45 (44.6)	3 (3.0)	3 (3.0)	NA	2 (2.0)	NA
Zhao G, et al. [85]	29 January 2021	Retrospective	China	36	51.24	13 (36.1)	23 (63.8)	6 (16.6)	6 (16.6)	NA	NA	NA
Zheng M, et al. [88]	19 March 2020	Cohort	China	68	47.13 (11–84)	36 (52.94)	32 (47.06)	3 (4.41)	3 (4.41)	NA	NA	NA
Zhou F, et al. [90]	11 March 2020	Retrospective, cohort	China	191	56.0 (46.0–67.0)	119 (62)	72 (38)	9 (4.71)	9 (5.0)	NA	7 (4.0)	NA
Zhou Z, et al. [91]	18 March 2020	Retrospective	China	254	50 (36–65)	115 (45.3)	139 (54.7)	66 (25.9)	46 (18.1)	3 (1.2)	36 (14.17)	NA
Yang W, et al. [80]	26 February 2020	Retrospective, cohort	China	149	45.11 ± 13.35	81 (54.36)	68 (45.63)	11 (7.38)	11 (7.38)	NA	2 (1.34)	NA
Zhang G, et al. [82]	9 April 2020	Retrospective, case series	China	221	55.0 (39.0–66.5)	108 (48.9)	113 (51.1)	80 (36.19)	25 (11.3)	5 (2.3)	NA	80 (36.2)
Wang R, et al. [67]	24 March 2020	Retrospective, descriptive	China	125	38.76 ± 13.799	71 (56.8)	54 (43.2)	50 (40.0)	50 (40.0)	NA	24 (19.2)	NA
Du R, et al. [23]	7 April 2020	Retrospective	China	109	70.7 ± 10.9	74 (67.88)	35 (32.1)	29 (26.6)	29 (26.6)	NA	NA	NA
Zheng T, et al. [89]	4 June 2020	Retrospective	China	1320	50 (40–57)	579 (43.9)	741 (56.1)	192 (14.54)	107 (8.1)	11 (0.8)	57 (4.3)	62 (4.7)
Zhao X, et al. [87]	29 April 2020	Retrospective	China	91	46.0	49 (53.8)	42 (46.2)	19 (12.1)	14 (15.4)	2 (2.2)	19 (12.1)	11 (12.1)
Zhang J-J, et al. [83]	18 February 2020	Retrospective	China	140	57 (25–87)	71 (50.7)	69 (49.3)	31 (22.3)	18 (12.9)	8 (5.8)	31 (22.3)	17 (12.2)
Xiao Y, et al. [77]	5 August 2020	Descriptive	China	90	61.0 (48.3–69.0)	51 (57)	39 (43)	37 (41.1)	8 (9.0)	6 (7.0)	37 (41.1)	22 (24)
Wei X, et al. [71]	18 July 2020	Retrospective, single centre	China	84	37 (24–74)	28 (33)	56 (66.6)	26 (30.9)	26 (30.9)	2 (2)	22 (26.1)	NA
Han J, et al. [30]	5 August 2020	Retrospective	China	120	45.4 (15.6)	43 (36)	77 (64.17)	7 (5.83)	7 (5.83)	NA	NA	NA
Jiang Y, et al. [35]	7 December 2020	Retrospective	China	495	42.24 ± 16.99	515 (41.5)	723 (58.4)	76 (15.3)	29 (5.85)	10 (2.02)	35 (7.0)	7 (1.4)
Lin L, et al. [41]	2 April 2020	Retrospective	China	95	45.3 ± 18.3	45 (47.4)	50 (52.6)	58 (61)	23 (24.2)	NA	17 (17.9)	17 (17.9)
Liu Y, et al. [43]	18 May 2020	Retrospective	China	148	56.5 ± 15.2	67 (45.2)	81 (54.7)	42 (28.3)	18 (12.16)	2 (1.34)	4 (2.7)	27 (18.2)
Luo S, et al. [47]	23 July 2020	Retrospective cohort	China	183	NA	102 (55.7)	81 (44.2)	183 (100)	68 (37.1)	65 (35.5)	119 (65.0)	180 (98.3)
Wang X, et al. [68]	14 April 2020	Retrospective	China	80	39 (32–48.5)	31 (38.75)	49 (61.25)	15 (18.75)	15 (18.75)	NA	NA	NA
Chen Q, et al. [19]	28 April 2020	Retrospective, single centre	China	145	47.5 ± 14.6	79 (54.5)	66 (45.5)	62 (42.75)	39 (26.8)	8 (5.5)	30 (20.6)	62 (42.75)
He S, et al. [31]	15 October 2020	Retrospective	China	267	57 (37–68)	116 (43)	151 (56.5)	20 (7)	20 (7)	NA	NA	NA
Hu C, et al. [32]	18 March 2021	Retrospective	China	32	NA	17 (53.1)	15 (46.8)	3 (9.4)	3 (9.4)	NA	NA	NA
Tu Y, et al. [65]	11 January 2021	Retrospective	China	74	68.0 (61.5–74.0)	53 (71.6)	21 (28.3)	24 (32.4)	24 (32.4)	2 (2.7)	5 (6.8)	NA
Wang Z H, et al. [70]	20 July 2020	Retrospective	China	59	67.4 ± 11.3	38 (64.4)	21 (35.6)	22 (37.3)	22 (37.3)	NA	4 (6.8)	11 (18.6)
Zheng F, et al. [81]	10 April 2020	Retrospective	China	161	45 (33.5–57)	80 (49.7)	81 (50.3)	17 (10.6)	17 (10.6)	NA	6 (3.7)	NA
Cai Q, et al. [15]	2 April 2020	Retrospective	China	298	47.5 (33–61)	145 (48.6)	153 (51.3)	9 (3.02)	9 (3.02)	NA	NA	NA
Duarte-Neto A, et al. [24]	22 May 2020	Case Series	Brazil	10	63 (33–83)	5 (50)	5 (50)	2 (20)	2 (20)	NA	NA	NA
Redd W, et al. [56]	22 April 2020	Multicentre, cohort	USA	318	63.4 ± 16.6	174 (54.7)	144 (45.3)	195 (61.3)	107 (33.7)	46 (14.5)	95 (29.8)	110 (34.8)
Chen A, et al. [18]	15 May 2020	Prospective, case–control	USA	340	46.89 ± 15.34	96 (28)	244 (71.7)	201 (59)	123 (36)	72 (21)	135 (39.7)	117 (34)
Cholankeril G, et al. [21]	28 April 2020	Retrospective	USA	207	49 (34–65)	104 (50.2)	103 (49.8)	70 (34.5)	22 (10.8)	14 (7.1)	22 (10.8)	NA
Ramchandran P, et al. [55]	29 June 2020	Retrospective, cohort	USA	31	57.6 ± 17.2	19 (61.2)	12 (38.7)	31 (20.6)	15 (10)	NA	6 (4)	NA
Nobel Y, et al. [51]	12 April 2020	Retrospective, case–control	USA	278	NA	145 (52)	133 (48)	97 (34.8)	56 (22.31)	NA	63 (25.09)	NA
Elmunzer B, et al. [25]	30 September 2020	Observational, cohort	USA	1992	60.1 ± 16.3	1128 (56.6)	864 (43.4)	1052 (53)	679 (34)	220 (11)	539 (27)	NA
Ferm S, et al. [26]	1 June 2020	Retrospective	USA	892	59 (47–72)	534 (59.8)	358 (40.1)	219 (24.6)	177 (19.8)	70 (7.8)	148 (16.6)	105 (11.8)
Renelus B, et al. [58]	4 September 2020	Retrospective	USA	734	66.1 ± 15.6	379 (51.6)	355 (48.4)	231 (31.5)	149 (20.3)	68 (9.26)	109 (14.9)	NA
Kang M, et al. [37]	13 July 2020	Retrospective	South Korea	118	61 (50–70)	52 (44.1)	66 (55.9)	54 (45.8)	54 (45.8)	NA	NA	NA
Banno A, et al. [14]	9 February 2021	Retrospective, observational	Japan	24	57.5 (49–68.8)	19 (79)	5 (20.8)	6 (25)	6 (25)	NA	NA	NA
Remes-Troche J, et al. [57]	21 May 2020	Cohort	Mexico	112	43.72 ± 15	81 (72.3)	31 (27.7)	23 (20.5)	20 (17.8)	11 (9.8)	8 (7.1)	NA
Namendys-Silva S, et al. [49]	21 October 2020	Multicentre observational	Mexico	164	57.3 ± 13.7	114 (69.5)	50 (30.4)	29 (17.6)	29 (17.6)	NA	NA	NA
Sulaiman T, et al. [64]	18 September 2020	Retrospective	Iraq	140	44.99 ± 16.81	100 (71.42)	40 (28.57)	78 (55.7)	41 (29.28)	42 (30)	31 (22.14)	40 (28.57)
Wolday D, et al. [72]	14 July 2021	Prospective, cohort	Ethiopia	751	37 (28-50)	480 (63.9)	14 (1.9)	76 (10.9)	39 (5.2)	44 (5.9)	76 (10.9)	NA
Aumpan N, et al. [6]	6 July 2020	Retrospective	Thailand	40	30.5 ± 9.2	18 (45)	22 (55)	12 (30)	6 (15)	2 (5)	2 (5)	7 (17.5)
Puah S, et al. [54]	5 April 2021	Prospective, multicentre	Singapore	60	44 (41–47)	37 (62)	23 (38.3)	10 (17)	10 (17)	NA	NA	NA
Jang J, et al. [34]	2 June 2020	Retrospective	South Korea	110	56.9 ± 17.0	48 (43.6)	62 (56.4)	11 (10)	11 (10.0)	NA	3 (2.7)	NA
Cheung K, et al. [20]	3 April 2020	Retrospective	Hong Kong	59	58.5 (43.5–68)	27 (45.7)	32 (54.2)	15 (25.42)	13 (22.0)	7 (11.9)	1 (1.7)	NA
Carvalho H, et al. [22]	4 January 2021	Case–control	France	1,188	65 (51.5–76)	663 (55.8)	524 (44.2)	202 (17.0)	202 (17.0)	NA	137 (10.6)	NA
Aghemo A, et al. [12]	10 May 2020	Retrospective	Italy	292	65 ± 14.1	199 (68.2)	93 (31.8)	69/245 (28.2)	69/255 (27.1)	NA	11/274 (4.0)	NA
Park S, et al. [53]	10 June 2020	Prospective	South Korea	46	26 (18–57)	21 (45.6)	25 (54.3)	16 (34.7)	7 (15.2)	5 (10.8)	1 (2.1)	1 (2.2)
Lo I, et al. [45]	15 March 2020	Retrospective	Macau	10	54 (27–64)	3 (30)	7 (70)	8 (80)	8 (80)	2 (20)	5 (50)	NA
Kashefizadeh A, et al. [38]	10 November 2020	Retrospective	Iran	53	58.4 ± 13.0	24 (45.3)	29 (54.7)	49 (80.8)	32 (61.5)	49 (80.8)	40 (76.9)	20 (38.5)
Hajifathalian K, et al. [28]	7 May 2020	Retrospective	USA	1059	61.1 ± 18.3	611 (57.7)	448 (42.3)	827 (78)	234 (22.1)	72 (6.8)	168 (15.9)	NA
Utku A, et al. [16]	17 August 2020	Cohort	Turkey	143	55.63 (mean)	77 (53.8)	66 (46.1)	31 (21.7)	31 (21.7)	NA	NA	NA
Kim C, et al. [39]	21 April 2021	Retrospective	South Korea	106	28 ± 9.3	46 (43.4)	60 (56.6)	7 (6.6)	7 (6.6)	NA	NA	NA
Shimamura Y, et al. [61]	16 July 2021	Retrospective	Japan	315	60 (41–74)	179 (57)	136 (43.1)	45 (14.0)	45 (14)	45 (14)	5 (2)	30 (10)
Sim B, et al. [62]	17 November 2020	Observational	Malaysia	5889	34.0 (24–51)	4221 (71.7)	1,668 (28.3)	298 (5.1)	298 (5.1)	NA	108 (1.8)	NA
Zhao W, et al. [93]	19 August 2022	Retrospective	China	208	53.5 ± 20.9	90 (44)	118 (56)	24 (12)	24 (12)	17 (8)	15 (7)	NA
Lee D-S, et al. [92]	25 May 2022	Retrospective	South Korea	46	60 (56–74)	22 (47.8)	24 (52.9)	25 (54)	25 (54)	28 (60.8)	11 (23.9)	NA
Delavari A, et al. [95]	10 March 2022	Retrospective	Iran	42,964	51.36 ± 19.61	22,854 (53.2)	20,110 (46.8)	6356 (14.7)	1198 (2.78)	688 (1.60)	1781 (4.14)	1,638 (3.81)
Belabbes F-Z, et al. [97]	9 September 2022	Retrospective, cohort	Morocco	154	NA	85 (55.2)	69 (44.2)	24 (15.6)	24 (15.6)	9 (5.8)	8 (5.2)	5 (3.3)
Sun Z, et al. [94]	20 January 2022	Retrospective	China	63	48.0 ± 21.2	39 (61.9)	24 (38.2)	9 (14.3)	9 (14.3)	NA	NA	NA
Chen D, et al. [96]	20 October 2022	Observational, cross-sectional	China	93	58.0 ± 12.1	46 (49.4)	47 (50.6)	65 (69.5)	27 (29.3)	NA	26 (27.7)	63 (67.9)

**Table 3:** COVID-19 infection with diarrhoea: summary of severe versus non-severe cases.

Authors	Date/Year of Publication	Study Site	Sample Size	Diarrhoea (N)	Severe Diarrhoea	Severe Total	Non-Severe Diarrhoea	Non-Severe Total	Comparison
Guan W, et al. [27]	28 February 2020	China	1099	42	10	173	32	926	Severe vs. non-severe
Li K, et al. [40]	29 February 2020	China	83	7	2	25	5	58	Severe vs. non-severe
Wang D, et al. [66]	7 February 2020	China	138	14	6	36	8	102	ICU vs. non-ICU
Wang Z, et al. [69]	16 March 2020	China	69	10	2	14	8	55	SpO_2_ ≥ 90% vs. SpO_2_ ≤ 90%
Xu X-W, et al. [79]	19 February 2020	China	62	3	3	33	0	29	Time since symptom onset >10 days vs. ≤10 days
Zheng M, et al. [88]	19 March 2020	China	68	3	1	13	2	55	Severe vs. mild
Zhang G, et al. [82]	9 April 2020	China	221	25	9	55	16	166	Severe vs. non-severe
Du R, et al. [23]	7 April 2020	China	109	29	15	51	14	58	ICU vs. non-ICU
Zhang J-J, et al. [83]	18 February 2020	China	140	18	9	57	9	82	Severe vs. non-severe
Han J, et al. [30]	5 August 2020	China	120	7	5	30	2	90	Severe vs. All (diarrhoea vs. no diarrhoea)
Liu Yu, et al. [43]	18 May 2020	China	148	16	8	47	8	68	Severe vs. non-severe
Chen Q, et al. [19]	28 April 2020	China	145	39	16	43	23	102	Severe vs. non-severe
Huang C, et al. [33]	24 January 2020	China	41	1	0	13	1	28	ICU vs. non-ICU
Zheng F, et al. [81]	March 2020	China	161	17	1	30	16	131	Severe vs. non-severe
Cai Q, et al. [15]	2 April 2020	China	298	9	4	58	5	240	Severe vs. non-severe
Nobel Y, et al. [51]	12 April 2020	USA	278	53	11	44	42	207	Hospital admission vs. ICU admission
Jang J, et al. [34]	2 June 2020	South Korea	110	11	1	23	10	87	Severe vs. non-severe
Puah S, et al. [54]	5 April 2021	Singapore	60	10	1	10	9	50	Severe vs. mild
Aumpan N, et al. [6]	6 July 2020	Thailand	40	6	4	10	2	30	ICU vs. non-ICU
Zhao W, et al. [93]	19 August 2022	China	208	24	4	20	20	188	Severe vs. mild
Chen D, et al. [96]	20 October 2022	China	93	27	20	51	7	42	Severe vs. moderate
Sun Z, et al. [94]	20 January 2022	China	63	9	6	24	3	39	Severe vs. mild
Lee D-S, et al. [92]	25 May 2022	South Korea	46	25	7	16	18	30	Severe vs. mild
Total			3800	405	145	876	260	2863	

## Data Availability

Data are available upon reasonable request.

## Supplementary Materials

The following supporting information can be downloaded at: https://www.mdpi.com/article/10.3390/tropicalmed8020084/s1, Figure S1: Pooled prevalence of overall gastrointestinal symptoms among 88 included studies; Figure S2: A pooled estimate of the prevalence of diarrhoea symptoms in COVID-19 infected patients; Figure S3: A pooled estimate of the prevalence of nausea and vomiting symptoms in COVID-19 infected patients; Figure S4: A pooled estimate of the prevalence of abdominal pain symptoms in COVID-19 infected patients; Figure S5: A pooled estimate of the prevalence of loss of appetite symptoms in COVID-19 infected patients; Figure S6: A funnel plot for publication bias for the severity of diarrhoea in COVID-19 infected patients; Figure S7: Forest plot demonstrating sub-groups analysis of COVID-19-infected patients’ diarrhoea in Asian versus non-Asian groups: title; Table S1: Newcastle–Ottawa scale (NOS) summary assessment of the risk of bias in 16 cohort studies; Table S2: Newcastle–Ottawa scale (NOS) summary assessment of the risk of bias in 2 case-control studies; Table S3: Newcastle-Ottawa scale (NOS) summary assessment of the risk of bias for a cross-sectional study; Table S4: National Institutes of Health (NIH) study quality assessment tool for risk of bias for case-series studies.
