# Supplementary material for: Severity of COVID-19 in Patients with Diarrhoea: A Systematic Review and Meta-Analysis
Source: Trop Med Infect Dis. 2023 Jan 26;8(2):84. doi: 10.3390/tropicalmed8020084 (PMC9966065; doi:10.3390/tropicalmed8020084)
Supplement: Supplementary file 1 [file tropicalmed-08-00084-s001.zip › tropicalmed-2126393-supplementary.pdf]

Supplementary Materials

Table captions

Table S1. Newcastle-Ottawa scale (NOS) summary assessment of the risk of bias in 16 cohort studies.
Table S2. Newcastle-Ottawa scale (NOS) summary assessment of the risk of bias in 2 case-control studies.
Table S3. Newcastle-Ottawa scale (NOS) summary assessment of the risk of bias for a cross-sectional study.
Table S4. National Institutes of Health (NIH) study quality assessment tool for risk of bias for case-series studies.

Figure captions

Figure S1. Pooled prevalence of overall gastrointestinal symptoms among 88 included studies.
Figure S2. A pooled estimate of the prevalence of diarrhoea symptoms in COVID-19 infected patients.
Figure S3. A pooled estimate of the prevalence of nausea and vomiting symptoms in COVID-19 infected patients.
Figure S4. A pooled estimate of the prevalence of abdominal pain symptoms in COVID-19 infected patients.
Figure S5. A pooled estimate of the prevalence of loss of appetite symptoms in COVID-19 infected patients.
Figure S6. A funnel plot for publication bias for the severity of diarrhoea in COVID-19 infected patients.
Figure S7. Forest plot demonstrating sub-groups analysis of COVID-19-infected patients' diarrhea in Asian *versus* non-Asian groups.

Table S1. Newcastle-Ottawa scale (NOS) summary assessment of the risk of bias in 17 cohort studies.

Study, year (ref.)	Selection				Comparability of cohorts	Outcome			Score (0-9)	Evidence of Quality
	Exposed cohort representative	Non exposed cohort selection	Exposure ascertainment	Outcome not present at the start		Assessment	Follow-up length	Follow up adequacy		
Cohort studies										
Guan W *et.al*, 2020 (1)	★		★	★	★ ★	★	★	★	8-9	good
Han J *et.al*, 2020 (2)	★	★	★	★	★ ★	★	★		8-9	good
Chen Q *et.al*, 2020 (3)	★		★	★	★ ★	★	★		7-9	good
Zheng M *et.al*, 2020 (4)	★		★	★	★ ★	★	★		7-9	good
Du R *et.al*, 2020 (5)	★		★	★	★ ★	★	★		7-9	good
Zhang J-J *et.al*, 2020 (6)	★		★	★	★ ★	★	★		7-9	good
Huang C *et.al*, 2020 (7)	★		★	★	★ ★	★	★		7-9	good
Zhao W *et.al*, 2022(8)	★		★	★	★ ★	★	★		7-9	good
Sun Z *et.al*, 2022(9)	★		★	★	★ ★	★	★		7-9	good
Liu Y *et.al*, 2020 (10)	★		★		★ ★	★	★		6-9	fair
Cai Q et.al, 2020 (11)	★		★	★	★	★	★		6-9	fair
Li K *et.al*, 2020 (12)	★		★	★	★ ★	★	★		6-9	fair
Wang Z *et.al*, 2020 (13)	★		★	★	★	★	★		6-9	fair
Lee D-S *et.al*, 2022 (14)	★	★	★ ★		★ ★ ★	★ ★	★		6-9	fair
**A**umpan N *et.al*, 2020 (15)	★		★		★	★	★		6-9	fair
Jang J *et.al*, 2020 (16)	★		★		★	★	★		5-9	fair
Zheng F*et.al*, 2020 (17)	★								5-9	fair

Table S2. Newcastle-Ottawa scale (NOS) summary assessment of the risk of bias in 2 case-control studies.

Study, year (ref.)	Selection				Comparability of cases and controls	Exposure			Score (0-9)	Evidence of Quality
	Definition of cases	Representativeness of the cases	Selection of Controls	Definition of controls		Assessment of exposure	The same method of ascertainment for cases and controls	Non-Response rate		
Case-control										
Nobel Y *et.al*, 2020 (18)	★	★	★	★	★★	★	★	★	9-9	good
Puah S, 2021 (51)	★					★	★		7-9	good

Scoring is given as follows; 3 or 4 stars in the selection, 1 or 2 stars in comparability, and 2 or 3 stars in outcomes are required for a “good” quality score. A “fair” quality score required 2 stars within the selection, 1 or 2 stars in comparability, and 2 or 3 stars in outcomes. A “poor” quality score indicates 0 or 1 star in the selection, 0 stars for comparability, or 0 or 1 star for outcomes.

Table S3. Newcastle-Ottawa scale (NOS) summary assessment of the risk of bias in a cross-sectional study.

Study, year (ref.)	Representativeness of the sample	Sample selection procedure	Non-response rate	Risk factor exposure assessment	Adjustment for confounders	Reliability of diagnosis	Assessment of outcome	Statistical test	Score (0-8)
Chen D *et.al*, 2022 (19)	Yes	Convenience	No	Yes	No	Yes	Yes	Yes	5

Scoring is given as follows; very good studies: 9-10 points, good studies: 7-8 points, satisfactory studies: 5-6 points and unsatisfactory studies: 0 to 4 points

Table S4. National Institutes of Health (NIH) study quality assessment tool for risk of bias for case-series studies.

Study, year (ref.)	1. Was the study question or objective clearly stated?	2. Was the study population clearly and fully described, including a case definition?	3. Were the cases consecutive?	4. Were the subject comparable?	5. Was the intervention clearly described?	6. Were the outcome measures clearly defined, valid, reliable, and implemented consistently across all studies?	7. Was the length of follow-up adequate?	8. Were the statistical method well described?	9. Were the result well described?	10. Grade AHRQ standard
Wang D *et.al*, 2020 (20)	Yes	Yes	Yes	NK	Yes	Yes	Yes	Yes	Yes	Good
Xu X-W *et.al*, 2020 (21)	Yes	Yes	NK	NK	Yes	Yes	Yes	Yes	Yes	Good
Zhang G *et.al*, 2020 (22)	Yes	Yes	NK	Yes	Yes	Yes	Yes	Yes	Yes	Good

AHRQ = Agency for Healthcare Research and Quality; GRADE = Grading of Recommendations, Assessment, Development, and Evaluation; NK= not known. Questions 1,6,7 must be present for AHRQ standards: “good” if all three factors are present, “fair” if two factor are present, and “poor” or “insufficient quality” if one factor is present.

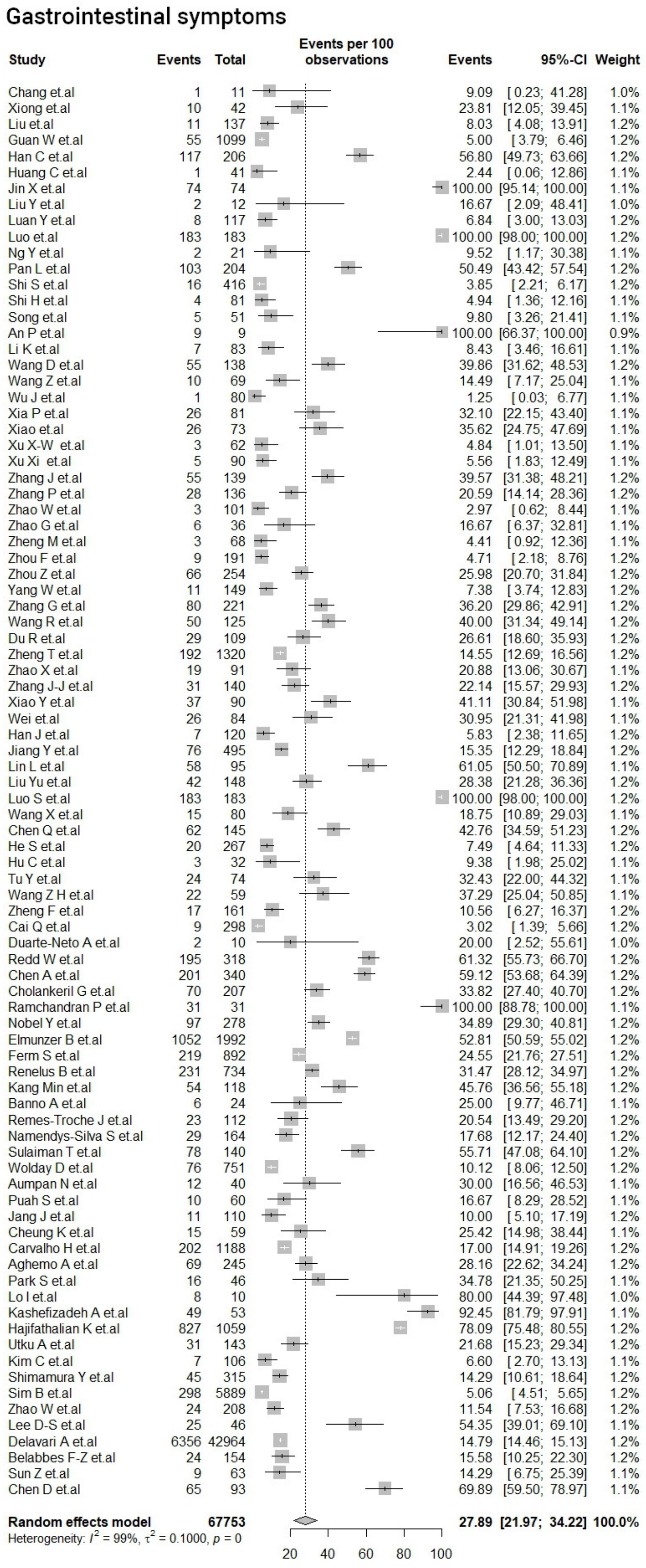

Figure S1. Pooled prevalence of overall gastrointestinal symptoms among 88 included studies.

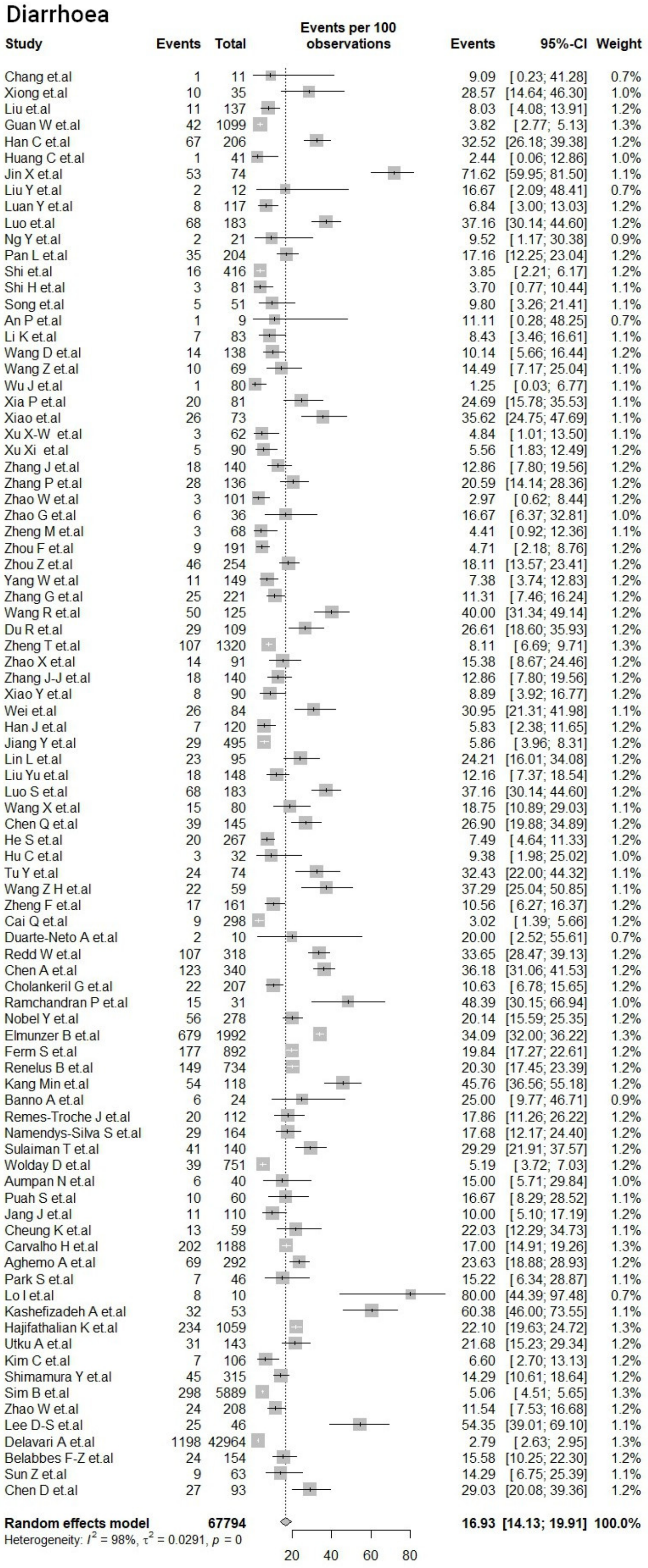

Figure S2. A pooled estimate of the prevalence of diarrhoea symptoms in COVID-19 infected patients.

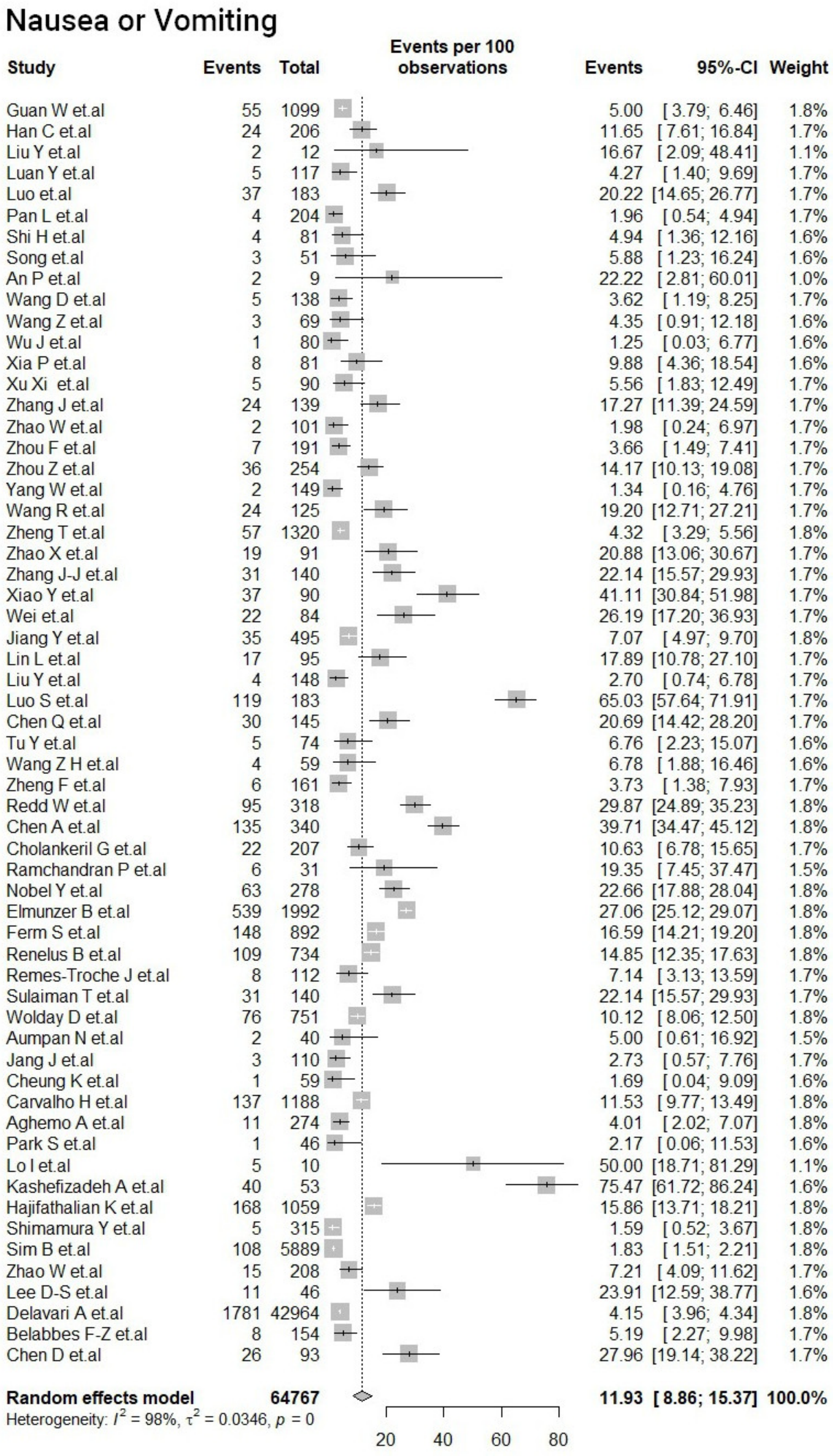

Figure S3. A pooled estimate of the prevalence of nausea and vomiting symptoms in COVID-19 infected patients.

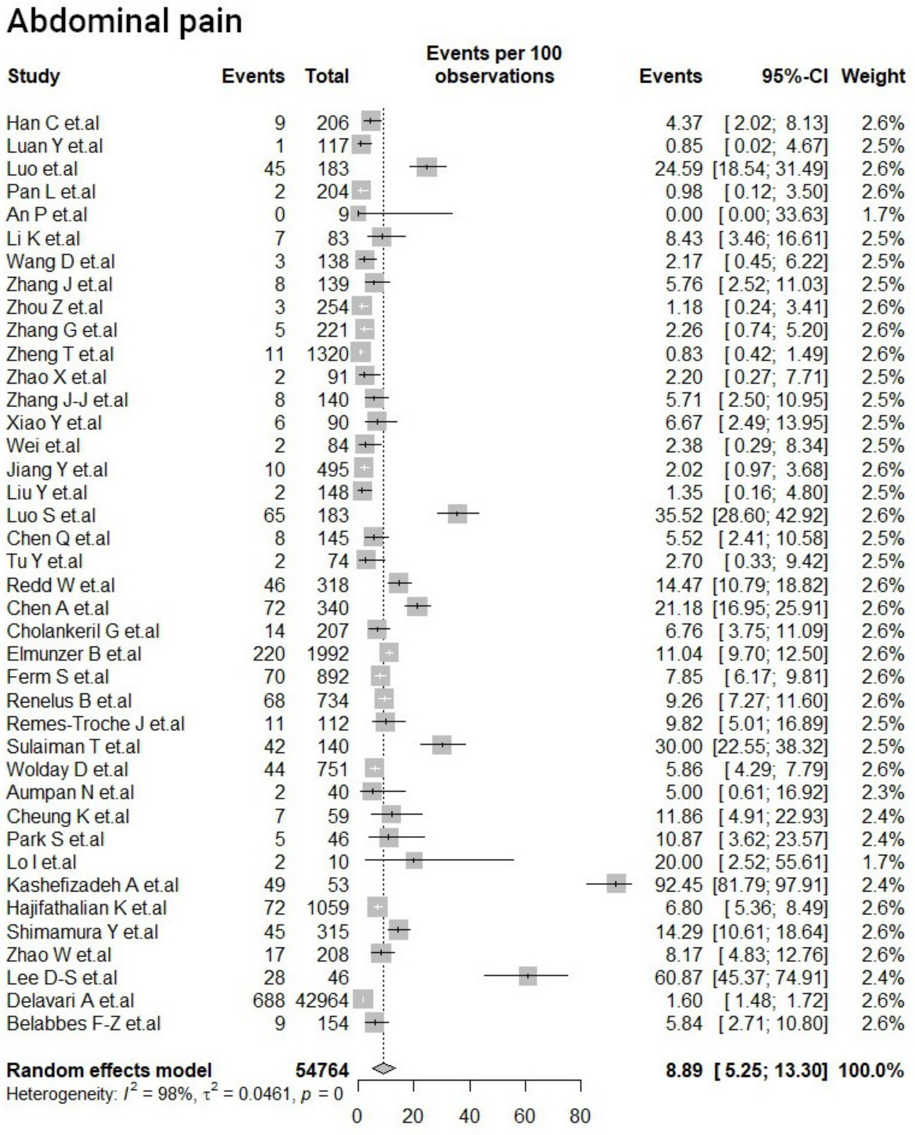

Figure S4. A pooled estimate of the prevalence of abdominal pain symptoms in COVID-19 infected patients.

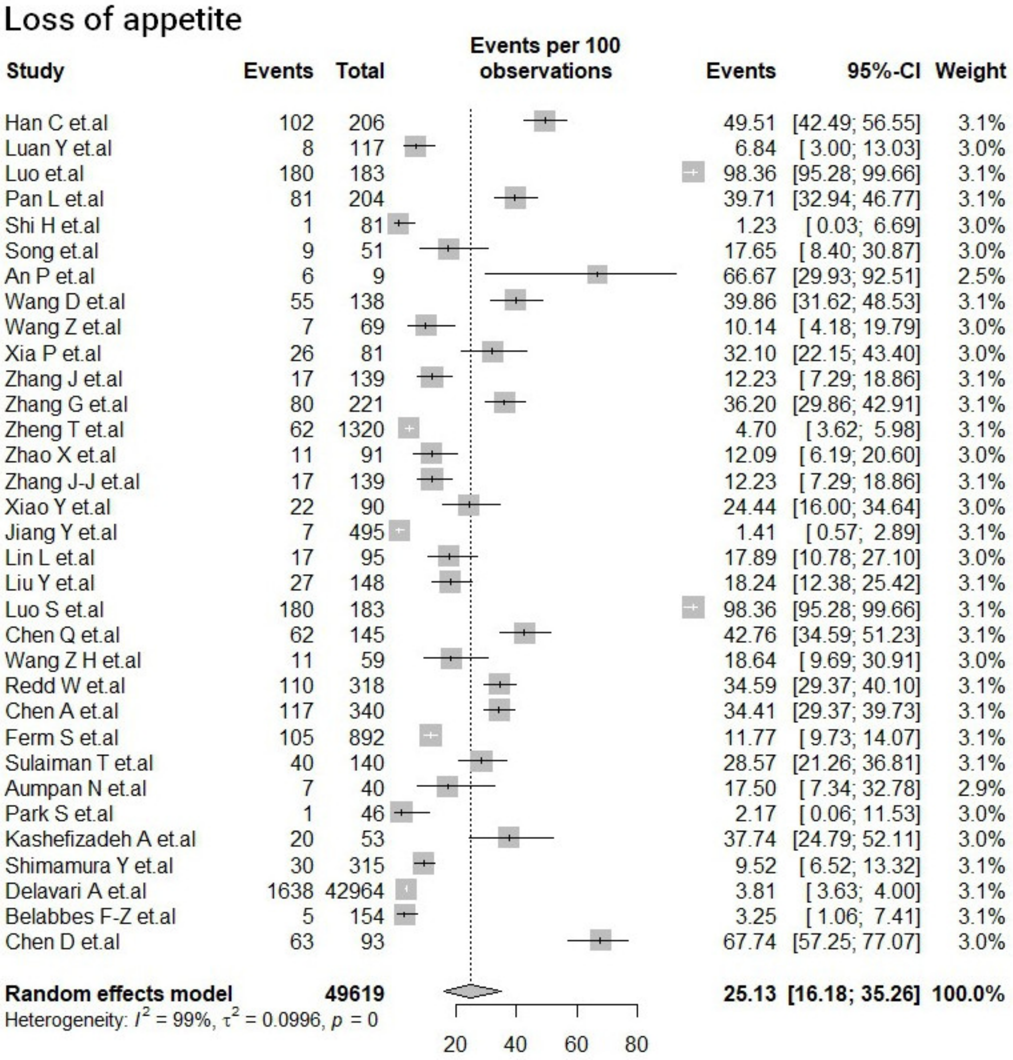

Figure S5. A pooled estimate of the prevalence of loss of appetite symptoms in COVID-19 infected patients.

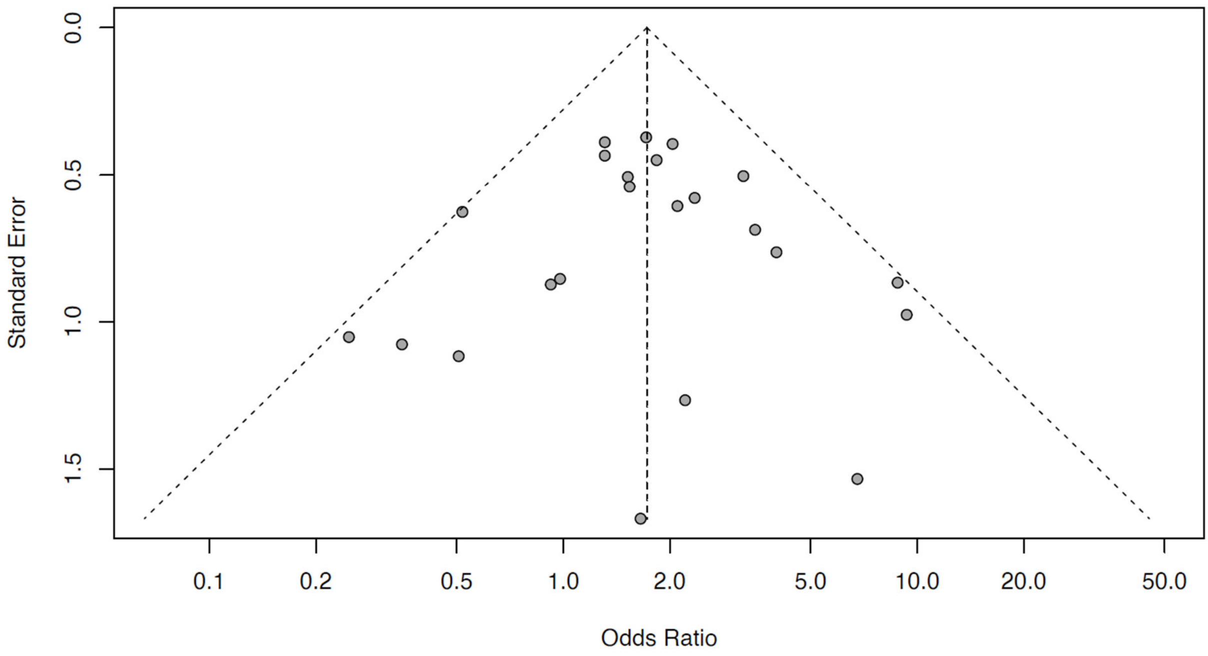

Figure S6. A funnel plot for publication bias for the severity of diarrhoea in COVID-19 infected patients.

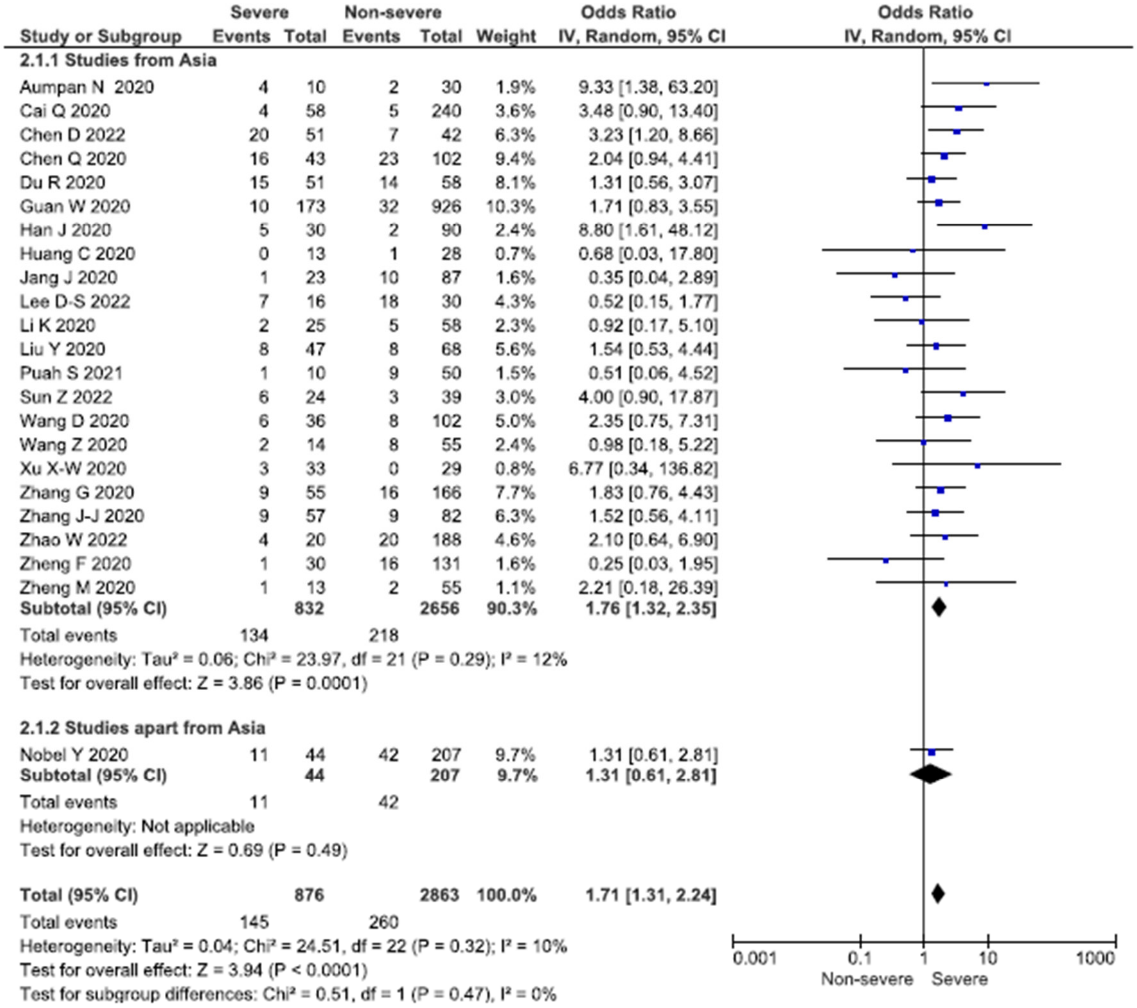

Figure S7. Forest plot demonstrating sub-groups analysis of COVID-19-infected patients' diarrhea in Asian *versus* non-Asian groups.

References

1. Guan W-J, Ni Z-Y, Hu Y, Liang W-H, Ou C-Q, He J-X, et al. Clinical Characteristics of Coronavirus Disease 2019 in China. New England Journal of Medicine. 2020;382(18):1708-20.
2. Han J, Gong H, Fu L, Chen P, Wang S, Yuan J, et al. Clinical and CT imaging features of SARS-CoV-2 patients presented with diarrhea. Journal of Infection. 2020;81(6):e33-e5.
3. Chen Q, Zheng Z, Zhang C, Zhang X, Wu H, Wang J, et al. Clinical characteristics of 145 patients with corona virus disease 2019 (COVID-19) in Taizhou, Zhejiang, China. Infection. 2020;48(4):543-51.
4. Zheng M, Gao Y, Wang G, Song G, Liu S, Sun D, et al. Functional exhaustion of antiviral lymphocytes in COVID-19 patients. Cellular & Molecular Immunology. 2020;17(5):533-5.
5. Du RH, Liu LM, Yin W, Wang W, Guan LL, Yuan ML, et al. Hospitalization and Critical Care of 109 Decedents with COVID-19 Pneumonia in Wuhan, China. Ann Am Thorac Soc. 2020;17(7):839-46.
6. Zhang J-J, Dong X, Cao Y-Y, Yuan Y-D, Yang Y-B, Yan Y-Q, et al. Clinical characteristics of 140 patients infected with SARS-CoV-2 in Wuhan, China. Allergy. 2020;75(7):1730-41.
7. Huang C, Wang Y, Li X, Ren L, Zhao J, Hu Y, et al. Clinical features of patients infected with 2019 novel coronavirus in Wuhan, China. The Lancet. 2020;395(10223):497-506.
8. Zhao W, Li Y, Xie R, Dong Y, Wei Y, Cheng C, et al. Real-World Evidence for COVID-19 Delta Variant's Effects on the Digestive System and Protection of Inactivated Vaccines from a Medical Center in Yangzhou, China: A Retrospective Observational Study. International Journal of Clinical Practice. 2022;2022:1-9.
9. Sun Z, Song Z-G, Liu C, Tan S, Lin S, Zhu J, et al. Gut microbiome alterations and gut barrier dysfunction are associated with host immune homeostasis in COVID-19 patients. BMC Medicine. 2022;20(1).
10. Liu Y, Xiang L, Deng K. Focusing on Gastrointestinal Symptoms in COVID-19 Is Far From Enough. Gastroenterology. 2021;160(4):1429-30.e2.
11. Cai Q, Huang D, Ou P, Yu H, Zhu Z, Xia Z, et al. COVID-19 in a designated infectious diseases hospital outside Hubei Province, China. Allergy. 2020;75(7):1742-52.
12. Li K, Wu J, Wu F, Guo D, Chen L, Fang Z, et al. The Clinical and Chest CT Features Associated With Severe and Critical COVID-19 Pneumonia. Invest Radiol. 2020;55(6):327-31.
13. Wang Z, Yang B, Li Q, Wen L, Zhang R. Clinical Features of 69 Cases With Coronavirus Disease 2019 in Wuhan, China. Clinical Infectious Diseases. 2020;71(15):769-77.
14. Lee DS, Kim JW, Lee KL, Jung YJ, Kang HW. Significance of digestive symptoms after COVID-19 vaccination: A retrospective single-center study. Am J Emerg Med. 2022;58:154-8.
15. Aumpan N, Nunanan P, Vilaichone RK. Gastrointestinal manifestation as clinical predictor of severe COVID -19: A retrospective experience and literature review of COVID -19 in Association of Southeast Asian Nations (ASEAN). JGH Open. 2020;4(6):1096-101.
16. Jang JG, Hur J, Choi EY, Hong KS, Lee W, Ahn JH. Prognostic Factors for Severe Coronavirus Disease 2019 in Daegu, Korea. Journal of Korean Medical Science. 2020;35(23).
17. Zg, F. Z, Tang W, Li H, Huang YX, Xie YL, et al. Clinical characteristics of 161 cases of corona virus disease 2019 (COVID-19) in Changsha. European review for medical and pharmacological sciences. 2020;24(6).
18. Nobel YR, Phipps M, Zucker J, Lebwohl B, Wang TC, Sobieszczyk ME, et al. Gastrointestinal Symptoms and Coronavirus Disease 2019: A Case-Control Study From the United States. Gastroenterology. 2020;159(1):373-5.e2.
19. Chen D, Ning M, Feng Y, Liu J. The early stage of COVID-19 pandemic: Gastrointestinal manifestations and liver injury in COVID-19 patients in Wuhan, China. Front Med (Lausanne). 2022;9:997000.
20. Wang D, Hu B, Hu C, Zhu F, Liu X, Zhang J, et al. Clinical Characteristics of 138 Hospitalized Patients With 2019 Novel Coronavirus–Infected Pneumonia in Wuhan, China. JAMA. 2020;323(11):1061.
21. Xu X, Yu C, Qu J, Zhang L, Jiang S, Huang D, et al. Imaging and clinical features of patients with 2019 novel coronavirus SARS-CoV-2. European Journal of Nuclear Medicine and Molecular Imaging. 2020;47(5):1275-80.
22. Zhang G, Hu C, Luo L, Fang F, Chen Y, Li J, et al. Clinical features and short-term outcomes of 221 patients with COVID-19 in Wuhan, China. Journal of Clinical Virology. 2020;127:104364.
